# Antibody Fragment and Affibody ImmunoPET Imaging Agents: Radiolabelling Strategies and Applications

**DOI:** 10.1002/cmdc.201800624

**Published:** 2018-11-15

**Authors:** Ruisi Fu, Laurence Carroll, Gokhan Yahioglu, Eric O. Aboagye, Philip W. Miller

**Affiliations:** ^1^ Department of Chemistry Imperial College London Exhibition Road South Kensington, London SW7 2AZ UK; ^2^ Comprehensive Cancer Imaging Centre, Department of Surgery and Cancer Imperial College London, Hammersmith Campus Du Cane Road London W12 0NN UK; ^3^ Antikor Biopharma Ltd. Stevenage SG1 2FX UK

**Keywords:** affibodies, antibody fragments, cancer imaging, PET imaging, radiolabelling

## Abstract

Antibodies have long been recognised as potent vectors for carrying diagnostic medical radionuclides, contrast agents and optical probes to diseased tissue for imaging. The area of ImmunoPET combines the use of positron emission tomography (PET) imaging with antibodies to improve the diagnosis, staging and monitoring of diseases. Recent developments in antibody engineering and PET radiochemistry have led to a new wave of experimental ImmunoPET imaging agents that are based on a range of antibody fragments and affibodies. In contrast to full antibodies, engineered affibody proteins and antibody fragments such as minibodies, diabodies, single‐chain variable region fragments (scFvs), and nanobodies are much smaller but retain the essential specificities and affinities of full antibodies in addition to more desirable pharmacokinetics for imaging. Herein, recent key developments in the PET radiolabelling strategies of antibody fragments and related affibody molecules are highlighted, along with the main PET imaging applications of overexpressed antigen‐associated tumours and immune cells.

## 1 ImmunoPET: Antibodies for PET Imaging

ImmunoPET, in its simplest terms, is the combination of an antibody, or related molecule, with a diagnostic positron‐emitting radionuclide for the purposes of imaging an associated antigen in vivo.^1^, ^2^ ImmunoPET is playing an important role in the diagnosis, staging and monitoring of treatment response in cancer. Although the combination of radionuclides with antibodies for imaging or therapy is not a new concept, recently there has been a resurgence in interest owing to advances in antibody engineering technology, the greater availability of diagnostic PET radionuclides and the development of new site‐specific chemical conjugation methods. The foundation of ImmunoPET is the matching of the antibody's ability to specifically engage a target at sub‐nanomolar affinities with the exquisitely high sensitivity of PET imaging. PET is a clinically‐based non‐invasive imaging technique that detects coincident gamma rays emitted from the positron decay/annihilation events from administered radiolabelled tracers.^3^, ^4^ The ability to detect very low levels of radioactivity via coincidence detection means that PET is incredibly sensitive, with only nanomolar amounts of a given PET tracer required for imaging. PET imaging is therefore a powerful clinical technique used to map the biodistribution of tracers and to quantify their uptake in vivo. The combination of an exceptionally high‐specificity/high‐affinity antibody molecule with a sensitive imaging technique such as PET should, in principle, produce a very powerful diagnostic tool that can complement other clinical imaging methods and interventions such as biopsied tissue and/or surgery. In practice, however, full antibody imaging agents based on whole, intact antibodies suffer some significant drawbacks mainly as a direct result of their large size (≈150 kDa). They have sluggish pharmacokinetics and resultantly long circulation times (up to 3 weeks); longer‐lived radionuclides (e.g., ^89^Zr, ^124^I) are therefore required for imaging. Such longer‐lived radionuclides are less ideal for clinical imaging due to higher associated radiation doses and longer wait times for imaging. The large size of intact antibodies typically results in clearance via the liver which can preclude imaging of liver disease. Slow blood clearance times and nonspecific binding of the tracer typically result in a higher background signal and therefore a decrease in the PET signal contrast; this ultimately leads to poorer image quality.^4^ As many full antibodies are also therapeutic agents they could in principle stimulate unwanted biological responses due to the interaction of their Fc regions with cell surface receptors, however, at the low concentrations typically used for PET imaging this is unlikely to happen. Ideally, the tracer should not perturb the biological system under study, therefore having an understanding of the imaging agent dose is important to ensure that it has minimal pharmacological or toxicological effects on the model, and also to ensure the highest possible contrast PET images. A recent study using an ^89^Zr‐labelled Cys‐diabody fragment for the preclinical imaging of CD4+ T‐cells demonstrated the importance of dose in obtaining high contrast images, and showed that when using their imaging agent a lower dose resulted in higher quality images.^5^

**Figure 1:**
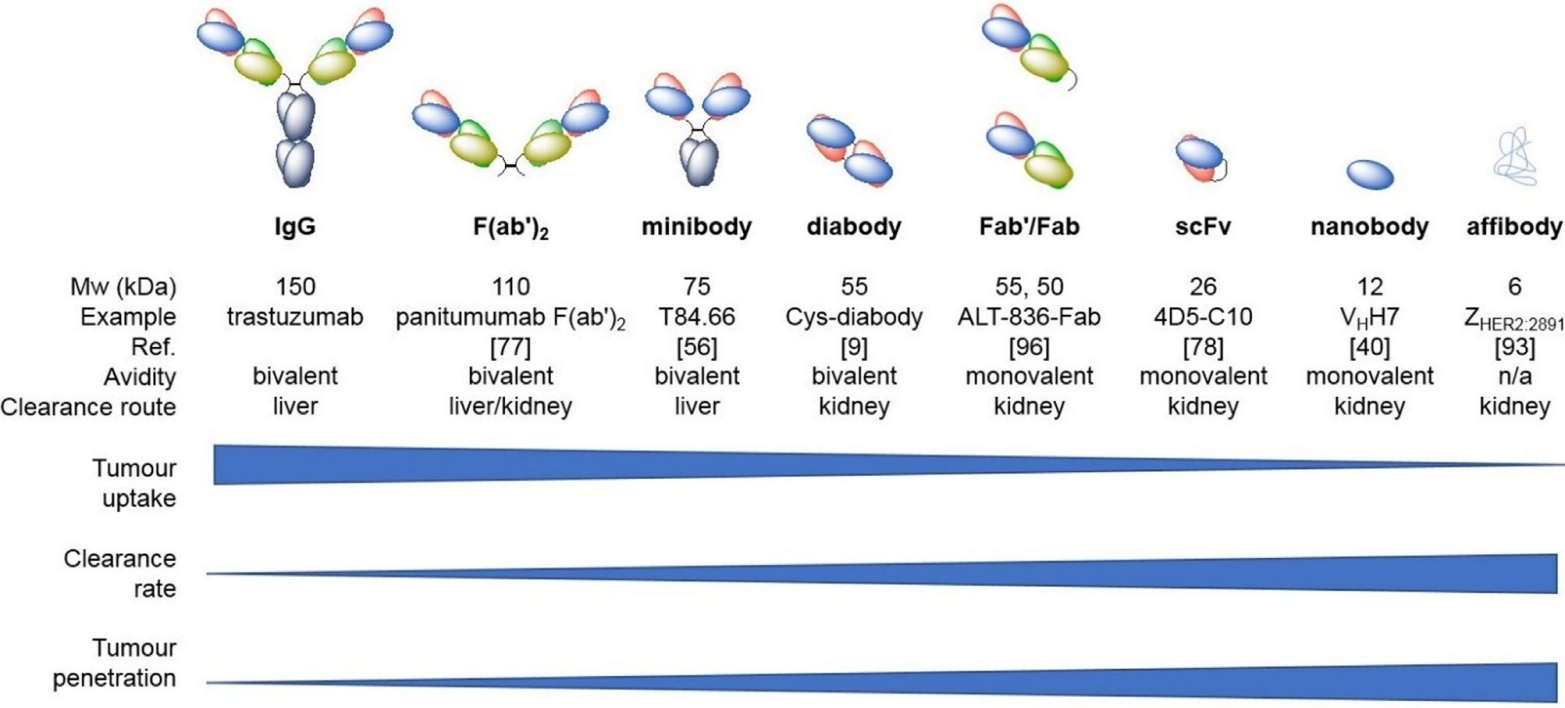
Summary of the key properties and examples of intact antibodies, antibody fragments and affibodies.

Antibody fragments are specifically engineered parts of antibodies that retain the desirable high affinities and specificities of full‐intact antibodies, but with more compatible pharmacokinetics for imaging.^6^ They essentially contain only the basic targeting and binding components. Despite their relative lack of complexity, the number of antibody fragments in clinical development is much smaller than that of intact antibodies.^7^, ^8^ To enable imaging they also need to contain sufficient functionality to attach a radionuclide. Typically, they range in size from 7 to 100 kDa, depending on the specific type of fragment (Figure 1). Antibody fragments have much shorter circulation times (hours rather than weeks), deeper penetration into tissue, are therefore better matched with the more clinically applied shorter‐lived PET radionuclides (e.g., ^18^F, ^68^Ga) and can enable same‐day imaging.^9^ This should, in theory, lead to improved images as blood clearance will be faster giving a better signal‐to‐noise ratio for the specifically bound radiolabelled fragment.^10^, ^11^ It may also be expected that fragments will be better tolerated by the subject, as they have been stripped of their variable domains, the part of the antibody that typically provoke much stronger immune responses. Absence of the Fc region also decreases the nonspecific binding of the fragment to other cells and can therefore improve image quality. In contrast to intact antibodies, antibody fragments, due to their much smaller sizes, are excreted via the renal system and are therefore not significantly metabolised or retained by the liver (Figure 1). Although antibody fragments do have the distinct advantages of better tumour penetration and faster blood clearance (Figure 1), they can have lower affinities and typically display lower overall tumour uptake than full antibodies.^12^

Affibody molecules are engineered proteins that have a 58 amino acid sequence folded into three alpha helices. They mimic monoclonal antibodies and antibody fragments with their high affinities and selectivities, however, they are much smaller (≈6–7 kDa, Figure 1) and chemically robust proteins that can tolerate higher temperatures and more extreme pH. Their small size, chemical robustness and high affinity (nanomolar range) make them excellent candidates to act as imaging probes.^13^ Their high affinities and short circulation times can result in high contrast PET imaging within hours of their administration.^14^ They can be efficiently selected for by phage display and produced on scale by recombinant techniques or by chemical methods using solid‐phase peptide synthesis.

Despite all the potential advantages of antibody fragments and affibodies, they do not in any way spell the end of full‐intact antibodies for imaging. The application of longer lived isotopes such as ^89^Zr (*t*_1/2_≈78 h) and ^124^I (*t*_1/2_≈100 h) for labelling intact antibodies can provide important continuous imaging information over longer time periods. The use of pre‐targeting strategies, whereby functionalised antibodies are given enough time to engage their target followed by administration of a positron emitting labelled molecule that will conjugate in vivo, enables the use of shorter‐lived PET radionuclides.^15^, ^16^ In this mini‐review, we highlight developments in the fragment‐based ImmunoPET area, discuss current strategies for radiolabelling antibody fragments and affibodies, and comment on the applicants for targeted imaging.

## 2 Antibody Fragments: A Design of the Times

The monoclonal antibody therapeutics market is set to be worth US$246 billion by 2024 with 74 approved antibody‐based drugs as of mid‐2017.^7^ This success is fuelling the development of the next generation of antibody‐based therapeutics and diagnostics. One area that has seen explosive growth has been in the development of alternative formats to intact antibodies especially in imaging and diagnostics driven by the inherent issues of both size and long serum residence time. Antibody fragments can be produced through either chemical/enzymatic digestion or genetic engineering.^17^, ^18^ The enzymatic digestion of intact antibodies with papain or pepsin is used to produce Fab′′ and (Fab′)_2_ fragments, these have one or two antigen‐binding regions, respectively but lack the Fc region that contains the heavy chain constant domains. Although Fab′′ fragments can exhibit rapid renal clearance and improved tumour penetration they cannot be prepared from all subclasses of antibodies through biochemical methods. Such production methods are laborious and require large quantities of starting intact antibody. The rapid development of antibody engineering technology has enabled the relatively easy production and isolation of the variable regions of antibodies, such as single‐chain variable region fragments (scFvs), and a range of engineered diabodies, minibodies and single‐domain antibody variants. Each of these variants has unique binding and functional characteristics. The various common fragments are shown in Figure 1. One of the most popular formats is the scFv.^19^ ScFv's have their V_H_ and V_L_ domains linked by a short flexible peptide chain between the C‐terminus of one Fv region to the N‐terminus of another. These fragments are small (26 kDa) and when the linker is at least 12 residues long are both monomeric and monovalent. In terms of design and engineering, a single gene sequence codes for the entire scFv. Shortening the peptide linker (<11 residues) between the V domains forces the scFv to self‐assemble, creating a bivalent diabody (55 kDa)^20^ and shortening the link even further (<3 residues) forces assembly into trivalent tribodies (80 kDa) and tetravalent tetrabodies (110 kDa). Genetically engineering an interchain disulfide bond between the two variable domains creates a disulfide stabilised Fv fragment (dsFv) without and with a peptide linker (scdsFv). These can address some of the stability and aggregation issues associated with scFvs. Minibodies are scFv‐CH3 fusion proteins where two scFvs are linked by a component of the heavy chain; these assemble into bivalent dimers (75 kDa). Bivalent single‐chain variable fragments (bi‐scFvs, 55 kDa) are engineered by linking two scFvs and have two different domains allowing them to bind to two different epitopes. The smallest known fragments that are still capable of selectively binding an antigen like an intact antibody are single domains, and are derived from either V_H_ or V_L_ regions or isolated from camelids. These single‐domain antibodies (sdAbs, 12–15 kDa), or nanobodies,^21^ have a number of distinct advantages over scFvs in terms of stability, ease of production and size. They are particularly amenable to applications such as radionuclide‐based imaging, which requires a combination of enhanced tissue penetration and rapid clearance. On an even smaller scale, affibodies (6–7 kDa) are engineered affinity proteins derived from the B‐domain in the immunoglobulin‐binding region of staphylococcal protein A through phage display.^13^ Affibodies seem ideally suited for ImmunoPET due to their small size, stability (extreme pH and temperature) and the presence of a unique C‐terminal cysteine for conjugation.^22^ However, their rapid clearance and decreased avidity for the target remain challenging.

## 3 Labelling Strategies and Applications

A range of common PET radionuclides have been used to label antibody fragments and affibodies (Table 1). Their physical properties—half‐life, decay characteristics and labelling chemistry—dictate both the types of fragments that can be labelled and how they are labelled. Key to the labelling of any tracer, including antibody fragments, is the appropriate matching of the radionuclide half‐life with the biological process under study to ensure that there is sufficient tracer accumulation to observe a signal. Because fragments have longer biological half‐lives than small molecule tracers (≈200–800 Da), tissue penetration is slower and clearance times are longer, thus radionuclides with *t*_1/2_ on the order hours are required. The positron yield and positron energy of the radionuclide affects the sensitivity and the image resolution, respectively. High positron yields as observed for ^11^C, ^19^F and ^68^Ga result in higher sensitivity, as the major decay pathway is via positron emission. Radionuclides with alternative decay pathways, such as ^64^Cu or ^89^Zr, may require larger doses to be administered to the subject in order to obtain the required level of signal. A low positron energy is desirable because this determines the distance the positron will travel in the body after decay, prior to annihilation. This is one of the physical limitations that determines the resolution of a PET image.

**Table 1:** PET radionuclides most commonly used for antibody fragment and affibody radiolabelling.

Radionuclide	Half‐life	β^+^ branching ratio [%]	Positron energy [MeV]	Production method
^11^C	20.4 min	99	0.97	^14^N(p,α)^11^C
^18^F	109.7 min	97	0.65	^18^O(p,n)^18^F
^68^Ga	67.7 min	89	1.90	^68^Ge/^68^Ga (generator)
^44^Sc	3.97 h	94	1.47	^44^Ca(p,n)^44^Sc or ^44^Ti/^44^Sc (generator)
^64^Cu	12.7 h	18	0.65	^64^Ni(p,n)^64^Cu
^89^Zr	78.4 h	23	0.91	^89^Y(p,n)^89^Zr
^124^I	100.2 h	23	1.54	^124^Te(p,n)^124^I

The longer lived PET radionuclides ^89^Zr and ^124^I have been used to radiolabel intact antibodies,^23^, ^24^ whilst the faster pharmacokinetics of antibody fragments have enabled the use of the shorter‐lived nuclides ^18^F, ^64^Cu, ^44^Sc and ^68^Ga for imaging. The fragment labelling strategy depends on both the particular isotope chosen and the available functionality on the particular fragment for conjugation. Because antibody fragments are large biomolecules and their tertiary structures are determined by many complex noncovalent bonds, extreme temperatures, pH and reducing conditions during the radiolabelling process can affect their structural integrity. Selecting an appropriate radiolabelling strategy is therefore key to ensuring that the affinity and specificity of the antibody fragment is retained. In addition, the radionuclide needs to be covalently bound to the fragment, and remain kinetically and thermodynamically stable over the time‐course of imaging. Degradation of the fragment in vivo with the formation of various radio‐metabolites can confound the signal giving poor quality images. As a result, intact antibodies and various antibody fragments are almost exclusively radiolabelled via indirect labelling routes that first involve the preparation of a radiolabelled “prosthetic” group or a bifunctional chelate complex (in the case of radiometals), that can then be conjugated to the fragment under much milder reaction conditions. Additionally, the position of the chelate on the fragment, type of chelate, number of chelators attached, and relative size of chelate compared to the fragment may affect the targeting of the antibody fragment. The conjugation of such chelators can affect the local charge or lipophilicity of the fragment and therefore its physicochemical properties. This is known to have a greater influence on the binding of smaller fragments and affibodies.^25^, ^26^

The options for radiolabelling antibody fragments and affibodies are more limited than for intact antibodies, as they are smaller and have fewer potential sites for conjugation. For example, site‐specific conjugation with modified glycans, commonly used for intact antibodies, is not possible for antibody fragments because the C_H_2 domain region is no longer present.^27^ The majority of routes to generating labelled fragments therefore exploit either the exposed and reactive primary amine groups on lysine residues or the thiol groups of cysteines. For example, activated esters such as *N*‐hydroxysuccinimide (NHS) esters will rapidly react with primary amines at room temperature under a mild pH 7–9 range. Functionalised isothiocyanate (SCN) groups can also be used to react with free amine groups generating stable thiourea conjugates. The abundance of lysine residues on a given fragment can, however, lead to nonspecific conjugation and a heterogeneously labelled product.^11^ To achieve site‐specific conjugation, and a more homogenously labelled product, disulfide bonds and cysteine residues can be targeted.^28^ Cysteine residues commonly form disulfide bonds within proteins. These disulfide bonds act as an inter‐chain linkage forming a bridge between the heavy and light chains of intact antibodies. The disulfide bridges within larger fragments such as F(ab′)_2_ or Fabs can be exploited to form free thiol groups for conjugation. Following a mild reduction, a pair of cysteine thiols are formed which can rapidly react with a maleimide containing label at pH 6.5–7.5 generating a thioether. On smaller antibody fragments, such as nanobodies, diabodies and scFv, there are no such inter‐chain disulfide bridges, however, free cysteine residues can be engineered into the fragment. For example, Olafsen et al. developed a diabody bearing a disulfide bond linkage in order to provide free thiol groups for further conjugation.^29^ Further functionalisation of free cysteine thiol or lysine amine groups is possible to enable even greater control of site‐specific conjugation.^30^ The conjugation of a range of reactive groups such as *trans*‐cyclooctenes, alkynes, azides and tetrazines to functionalised amine or thiol residues to facilitate click reactions of complementary radiolabelled precursors can further improve the rate, efficiency and versatility of the labelling.^31^ Similar conjugation methods have been used to radiolabelling of affibodies. Because affibody molecules can be prepared via well‐known chemical peptide synthesis chelators, fluorescent dyes or radiolabelled groups can be introduced site‐specifically on the protein sequence.^13^ Such site‐specific labelling is typically achieved either via chelation of a radiometal, where the chelator has been introduced at a specific site in the sequence, or via reaction of an incorporated cysteine residue with an appropriately radiolabelled maleimide group, for example. The site‐specific labelling of such affibodies and fragments is important to ensure that the site of the radiolabel or chelator does not impact the binding, and also to ensure that the fragment is homogenously labelled in a single position and well‐characterised to better enable clinical translation.

ImmunoPET is playing an important role in cancer diagnosis, staging and monitoring of treatment response. Conventional biopsy detection methods are invasive and can cause significant discomfort to the patient. Biopsies can also suffer from diagnostic inaccuracies due to heterogeneous nature of receptors within a tumour mass. PET imaging by comparison is minimally invasive and can provide more accurate quantitative information about the primary tumour mass and secondary lesions. Relative to [^18^F]FDG PET, which monitors metabolic uptake, ImmunoPET provides biomarker information on a disease by directly targeting specific receptors. A number of recent studies have shown the potential of antibody fragment‐based ImmunoPET for detecting a broad range of diseases. The most common application to date has been the imaging of overexpressed antigen‐associated tumours; the human epidermal growth factor receptor (HER1, HER2 and HER3) being the most widely investigated. The detection of T‐cells have also been investigated by specifically targeting T‐cell receptors for the detection of inflammatory related conditions such graft‐versus‐host disease (MHC class II), inflammatory bowel disease (CD4) and inflammatory responses to hematopoietic stem cell transplant (CD4, CD8). Cardiovascular diseases, such as atherosclerosis, have also been targeted and evaluated by detecting the vascular cell adhesion molecule (VCAM‐1). An emerging area of ImmunoPET imaging is the direct targeting of pathogen specific antigens on viruses, bacteria and fungi. The growing area of antibody drugs also makes ImmunoPET an excellent diagnostic companion for monitoring the efficacy of these antibody‐based treatments.

### 3.1 Non‐metals: labelling with ^18^F, ^124^I and ^11^C

**Scheme 1:**
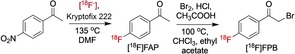
Typical ^18^F‐labelling synthetic route used to prepare [^18^F]FPB via 4‐[^18^F]fluoroacetophenone ([^18^F]FAP) followed by a bromination step. [^18^F]FPB can be used as labelling agent for conjugation to amine groups of lysine residues on antibody fragments.

**Figure 2:**
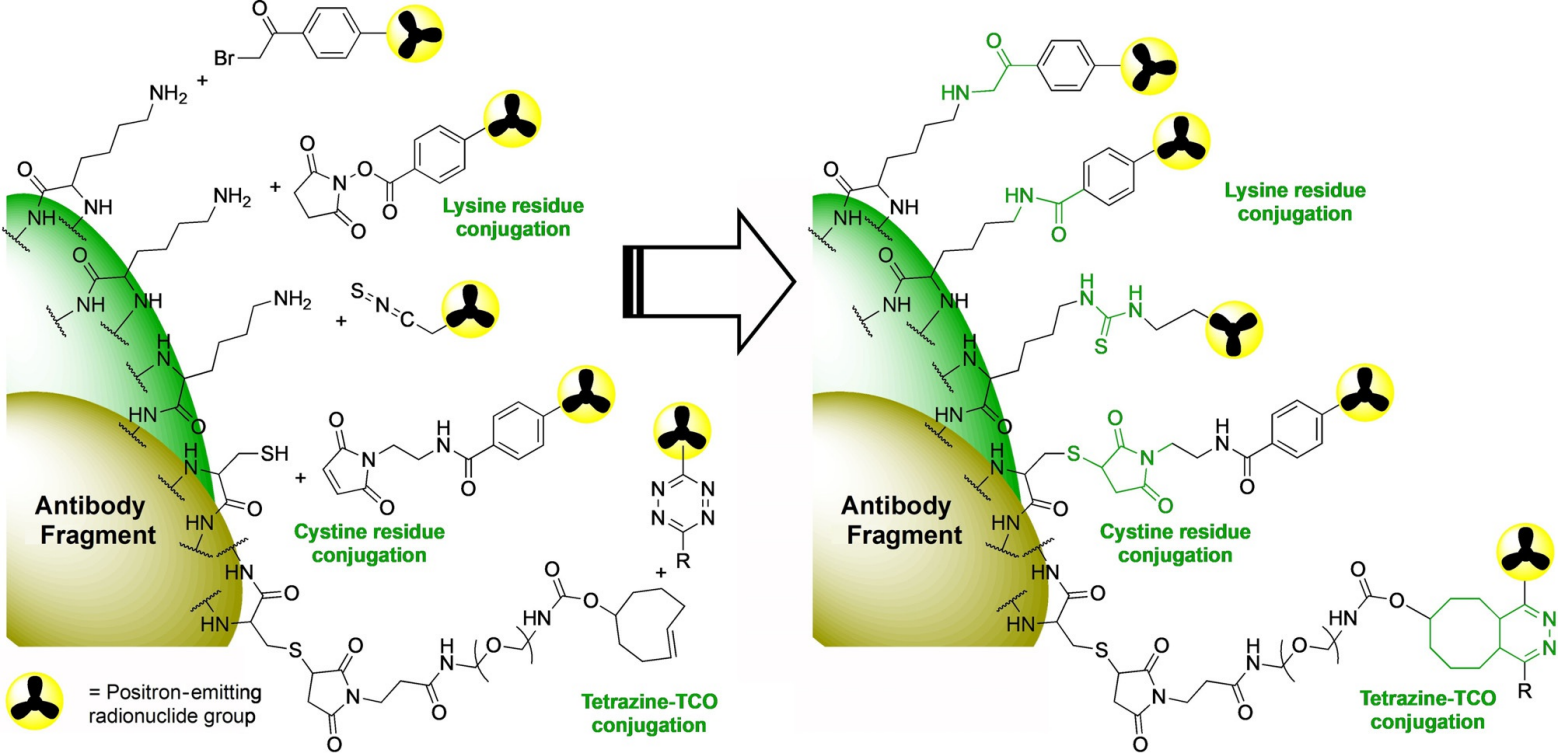
A selection of the common bioconjugation methods that have been used to radiolabel lysine and cysteine residues on antibody fragments or affibody molecules.

**Scheme 2:**
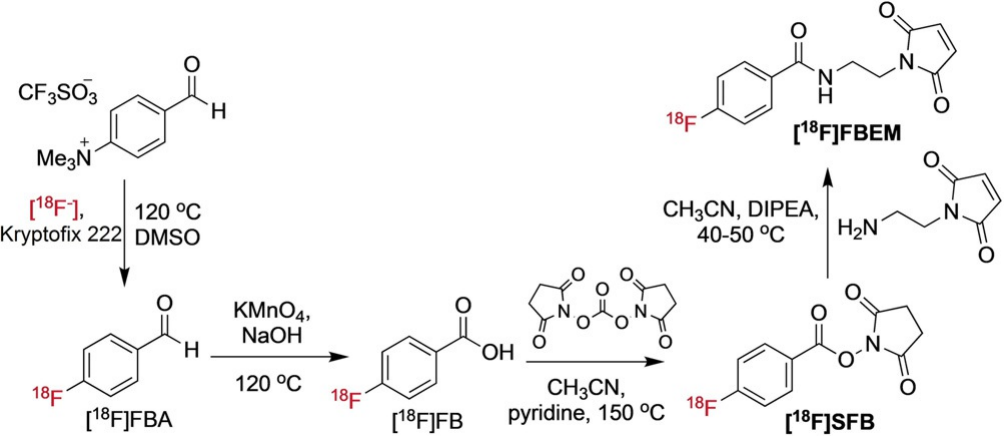
^18^F‐Labelling synthetic routes commonly used to prepare [^18^F]SFB and [^18^F]FBEM. [^18^F]SFB is typically prepared via ^18^F‐fluorination of a 4‐formyl‐trimethylanilinium triflate to give 4‐[^18^F]fluorobenzaldehyde ([^18^F]FBA) which is oxidised to 4‐[^18^F]fluorobenzoic acid ([^18^F]FB) and then reacted with *N*,*N*‐disuccinimidyl carbonate. [^18^F]FBEM can be generated from [^18^F]SFB via reaction with *N*‐(2‐aminoethyl)maleimide. [^18^F]FBEM is used to label free thiol groups on cysteine residues, and [^18^F]SFB can be used to conjugate to amine groups of lysine residues.

Fluorine‐18 is the most widely used PET radionuclide owing to its high positron yield, low positron energy, approximate two hour half‐life and routine cyclotron production via proton bombardment of [^18^O]H_2_O to generate [^18^F]fluoride. The short ∼110 min half‐life does, however, restrict the type of antibody fragment that can be considered for labelling. Furthermore, the high temperatures and nonaqueous conditions are typically used to incorporate [^18^F]fluoride into organic molecules are not compatible with the direct labelling of antibodies. There are, however, an array of reactions that can be exploited to indirectly radiolabel antibody fragments using various ^18^F‐prosthetic groups, thus avoiding the harsh direct labelling conditions. The caveat of prosthetic group labelling is the additional time and complexity required to prepare and purify these groups for further conjugation. Several preliminary studies of ^18^F‐labelling of F(ab′)_2_ fragments were achieved via conjugation reactions of lysines with *para*‐[^18^F]fluorophenacyl bromide ([^18^F]FPB, Scheme 1 and Figure 2)^32^ and the NHS activated ester *N*‐succinimidyl‐4‐[^18^F]fluorobenzoate ([^18^F]SFB, Scheme 2 and Figure 2).^33^ More recently diabodies^9^ and scFvs^34^ have been labelled using [^18^F]SFB. N‐2‐(4‐[^18^F]fluoro‐benzamido)ethyl]maleimide ([^18^F]FBEM, Scheme 2) is also an effective reagent for tagging thiol groups that has been used to develop a HER2‐binding cysteine rich affibody molecule.^35^ The presence of cysteine in the C‐terminus of the HER2 affibody molecule Z_HER2:2891_ has been exploited for labelling by three methods: silicon‐fluoride acceptor approach [^18^F]SiFA, [^18^F]AlF‐NOTA, and 4‐[^18^F]fluorobenzaldehyde ([^18^F]FBA); [^18^F]FBA was the favoured candidate for further development and characterisation in mouse models of breast cancer.^36^, ^37^

**Scheme 3:**
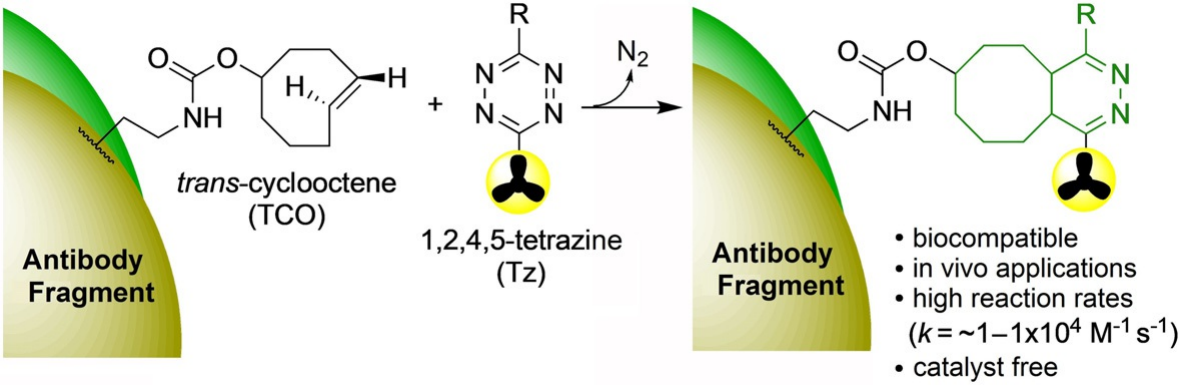
The inverse electron demand Diels–Alder (IEDDA) reaction using a radiolabelled 1,2,4,5‐tetrazine (Tz) and fragment linked *trans*‐cyclooctene (TCO) molecule has favourable characteristics for radiolabelling biomolecules (*k*=rate constant).

**Scheme 4:**
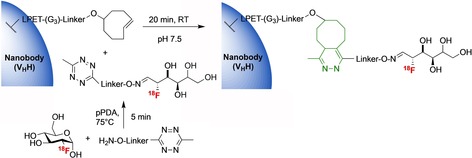
Reaction [^18^F]FDG with an aminooxy‐modified tetrazine to generate a ^18^F‐labelled Tz. Reaction of the ^18^F‐labelled Tz with a TCO‐modified single‐domain antibody fragment (V_H_H) for 20 min generates an ^18^F‐radolabelling nanobody following size‐exclusion chromatography.^40^

More recent labelling strategies have focused on conjugation reactions based on click chemistry that occur more efficiently and at much faster rates. This has been more widely adopted for affibody labelling where reactive cysteine residues can be incorporated into the sequence that can enable site‐specific labelling. A wide range of click reactions have been investigated for the bioconjugation of radiolabelled prosthetic groups to biomolecules, the most common approaches being: 1,3‐dipolar azide‐alkyne cycloaddition catalysed with copper, strain‐promoted azide‐alkyne cycloaddition (SPAAC), Staudinger ligation, and the inverse electron demand Diels–Alder (IEDDA).^31^ The IEDDA reaction,^38^ using a 1,2,4,5‐tetrazine (Tz)/*trans*‐cyclooctene (TCO) pair, has risen in prominence to become a powerful and convenient route for both modifying and labelling proteins. This [4+2] cycloaddition, which eliminates a molecule of dinitrogen and generates a new six‐membered ring (Scheme 3), fulfills a number of key criteria in terms of rapid reaction rates, selectivity and biocompatibility which are essential for radiolabelling applications. Since the first application of Tz/TCO for ^18^F‐labelling,^39^ a range of ^18^F‐Tz‐ and TCO‐labelled molecules have been reported to facilitate protein and peptide labelling.^38^ A recent elegant example of this Tz/TCO approach was used to radiolabel a nanobody. An aminooxy‐tetrazine was first reacted with [^18^F]2‐deoxy‐2‐fluoroglucose ([^18^F]FDG) to generate the ^18^F‐labelled tetrazine (Scheme 4).^40^ Reaction with [^18^F]FDG is a convenient way to introduce ^18^F, as it is produced daily at many PET centers and thus is both readily available and avoids more complicated labelling methods. The nanobody (V_H_H) was modified at its C‐terminus with a sortase‐recognition motif that was site‐specifically modified with a (Gly)_3_‐TCO unit. Reaction of the [^18^F]FDG‐tetrazine with the TCO modified nanobody enabled site‐specific labelling within a 20 min time frame.

**Scheme 5:**
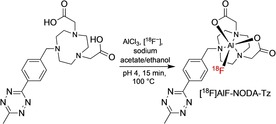
Preparation of [^18^F]AlF‐NODA‐Tz via reaction of the NODA‐functionalised tetrazine with [^18^F]{AlF}^2+^, generated in situ from AlCl_3_ and ^18^F^−^.^36^, ^42^, ^43^

**Figure 3:**
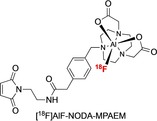
[^18^F]AlF‐NODA‐MPAEM has been used to radiolabel cysteine‐rich fragments.^36^, ^42^, ^43^

**Figure 4:**
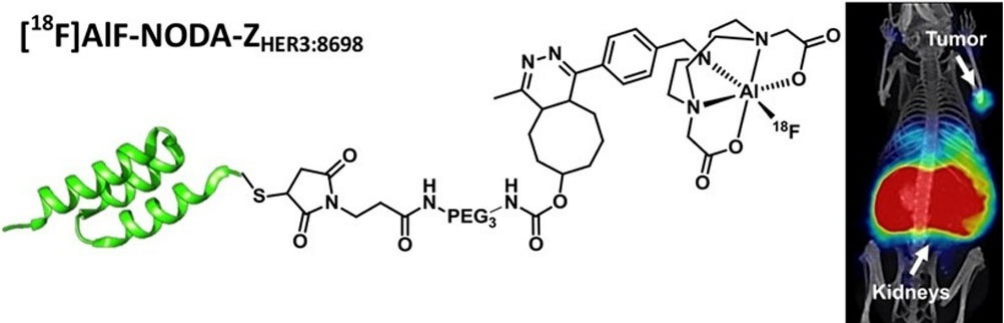
[^18^F]AlF‐NODA‐Z_HER3:8698_ affibody and PET/CT image showing uptake in MCF‐7 tumour‐bearing mice. Figure reproduced from Ref. 43 with permission; Copyright ©2016, American Chemical Society.

**Scheme 6:**
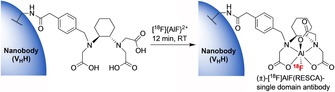
Room temperature chelation of [^18^F]{AlF}^2+^ to generate a radiolabelled [^18^F]AlF‐RESCA nanobody.^44^

Aluminum chelate complexes have recently been adapted for ^18^F‐labelling of antibody fragments, and are proving to be a very effective strategy. The aluminum‐[^18^F]fluoride ([^18^F]AlF) method, originally developed by McBride et al.,^41^ involves the reaction of a bifunctional chelator with [^18^F]AlF (generated in situ from ^18^F^−^ and AlCl_3_), followed by a conjugation step. Alternatively, the chelate can be conjugated to the vector molecule first, followed by direct [^18^F]AlF labelling. Typically, [^18^F]AlF is chelated to functionalised 1,4,7‐triazacyclononane‐1,4,diacetic acid (NODA) derivate at 100 °C for 15 min at pH 4 (Scheme 5, Figure 3). The key advantages of the [^18^F]AlF method are the improved efficiencies (RCY and molar activities) and simplification of the labelling process. NODA route has been used to successfully label a range of F(ab′)_2_, Fab, diabody and affibody fragments using both maleimide and tetrazine functionalised chelates (Figure 4).^36^, ^42^, ^43^ The elevated temperatures required to effect this chelation can affect the integrity of proteins if a direct labelling protocol is used. Recently, Bormans and co‐workers have overcome this issue with their (±)‐H3RESCA chelator (Scheme 6).^44^ They were able to synthesise [^18^F]AlF labelled nanobodies and affibodies at room temperature that had been prefunctionalised with their chelator, achieving RCYs similar to those of previously reported NOTA variations.

Enzymatic labelling methods have also recently been used for labelling Fab fragments. The lipoic acid ligase (LplA) enzyme was used to catalyze the site‐selective ligation of the [^18^F]fluorooctanoic acid ([^18^F]FA) substrate to a lysine residue on a signature peptide sequence on the antibody fragment (2G10) which has a high affinity for urokinase plasminogen activator receptor (uPAR or CD87).^45^ The labelling method was found to be fast and high yielding under aqueous conditions at 30 °C, and did not to affect the immunoreactivity of the fragment. However, [^18^F]FA is time consuming to prepare (≈180 min) and the methods needed to generate appropriately tagged fragments may ultimately limit this labelling method.

**Scheme 7:**
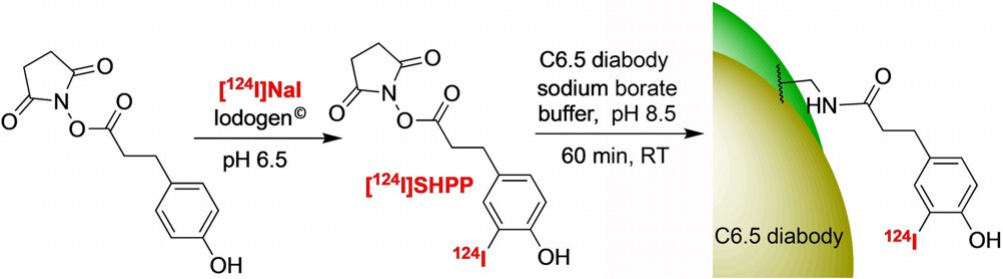
Radioiodination of a diabody fragment using [^124^I]SHPP.^50^

^124^Iodine (*t*_1/2_≈4 days) is a longer lived radionuclide that is well matched for the labelling of antibodies and larger fragments. Labelling with ^124^I is typically achieved via electrophilic substitution reactions with suitable leaving groups or activated phenyl rings to generate a covalent ^124^I‐carbon bond.^24^ Iodination reagents, such as Iodogen and chloroamine‐T, are able to oxidise [^124^I]NaI to generate ^124^I^+^ in situ which can then iodinate a suitability activated aromatic rings. The method is sufficiently mild to directly radiolabel proteins at low temperatures, and is frequently used to label the phenol ring of tyrosine. Such radioiodinations cannot however be used to label specific phenolic or tyrosine groups within the protein. Several minibodies for the imaging of PCSA and CD20 have been developed by Wu and co‐workers,^46^, ^47^, ^48^, ^49^ wherein they compared the performance of ^124^I iodinated fragments to that of ^89^Zr and ^64^Cu versions. Iodine labelled fragments are more susceptible to degradation and deiodination in vivo, releasing free [^124^I]iodide or [^124^I]iodotyrosine. This can lead to loss of signal in the region of interest over time. However, because iodine is able to diffuse out of the tissue, a lower background signal can lead to improved images relative to radiometal‐based tracers. Alternatively, a number of ^124^I‐prosthetic groups have also been developed that are suitable for mild and selective protein labelling, one example is *N*‐succinimidyl‐3‐(4‐hydroxy‐5‐[^124^I]iodophenyl)propionate ([^124^I]SHPP), which can react with the amines of lysine groups (Scheme 7). This route has been used to radiolabel a HER2 targeting diabody; however, the [^124^I]SHPP labelling method was shown to decrease the immunreactivity of this fragment relative to the ^124^I labelled version using the Iodogen method.^50^ This likely depends on the specific macromolecule as other examples exist in the intact antibody literature wherein the [^124^I]SHPP labelling method is preferred.^51^

The anti‐HER2 affibody Z_HER2:342_ has been labelled with ^124^I using a similar type of prosthetic group, *N*‐succinimidyl‐*para*‐[^124^I]iodobenzoate ([^124^I]SPIB).^52^ In a comparative study with [^124^I]trastuzumab, the total uptake radioactivity of [^124^I]trastuzumab in tumours was found to be higher than [^124^I]SPIB‐Z_HER2:342_, however, tumour‐to‐organ ratios were lower. [^124^I]SPIB‐Z_HER2:342_ showed a rapid clearance of radioactivity from blood and organs, and therefore gives better contrast than the intact antibody. The inherent limitations of ^124^I include low positron yield which affects sensitivity, high energy positron emission giving lower resolution and higher gamma emissions which increase patient doses. Even with these limitations longer‐lived tracers, such as ^124^I, continue to be important for PET imaging. The pairing of imaging isotopes such as ^124^I with a matched therapeutic partner such as ^131^I (for radiotherapy) will also become more important for combined imaging and therapy.

**Scheme 8:**
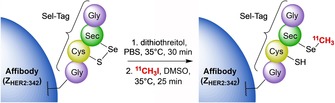
^11^C‐methylation of an affibody molecule using the Sel‐Tag method and ^11^CH_3_I.

Despite its short 20 min half‐life, carbon‐11 has been used to radiolabel a HER2 targeting affibody and a scFv. The affibody labelling route exploited a C‐terminal selenocysteine tetrapeptide Sel‐Tag, that enabled site‐specific labelling using the common ^11^C‐labelling reagent, [^11^C]methyl iodide within 45 min (Scheme 8).^53^ This labelling method, using the shorter ^11^C half‐life, may be promising for the rapid and repeated monitoring of HER2 expression levels in tumours, and also for monitoring of responses to therapeutic treatment over time using lower doses of radioactivity. These ^11^C‐labelled affibodies displayed clear tumour‐targeting, rapid blood and non‐target tissue clearance, which enabled visualisation of the tumours after only 30 min. A cell‐free synthesis has also been used to prepare ^11^C‐labelled anti‐human epidermal growth factor receptor variant III (EGFRvIII) scFv (MR1‐1).^54^ By adding l‐[^11^C]methionine, a clinically used PET tracer, to the cell‐free protein synthesis of MR1‐1, a [^11^C]MR1‐1 version was obtained in high radiochemical purity. Although proven viable for preclinical work, the application of such ^11^C labelled antibody fragments for human studies could prove challenging owing to the short half‐life.

### 3.2 Radiometals: labelling with ^64^Cu, ^68^Ga, ^89^Zr and ^44^Sc

Radiometals with longer half‐lives, such as ^64^Cu (*t*_1/2_=12.7 h) and ^111^In (*t*_1/2_=2.8 days), have been used for many years for the labelling and imaging of intact antibodies.^55^ The half‐life of ^64^Cu also makes it suitable for imaging larger fragments such as minibodies, (Fab′)_2,_ Fab and diabodies that have slower clearance rates. Key pioneering studies of antibody fragments for PET imaging have been conducted using ^64^Cu.^29^, ^56^ 1,4,7,10‐tetraazacyclododecane‐1,4,7,10‐tetraacetic acid (DOTA) and 1,4,7‐triazacyclononane‐1,4,7‐triacetic acid (NOTA) chelation methods have been the mainstay for ^64^Cu labelling of antibody fragments.^57^, ^58^, ^59^, ^60^ The 12.7 h half‐life relieves the time pressures for labelling, permitting extended reaction times of ≈60 min to ensure complete chelation of DOTA derivatives. ^64^Cu‐DOTA labelled antibodies and antibody fragments have shown high circulation radioactivity and nonspecific uptake in kidney, liver, and spleen.^29^, ^61^ This is partially due to competition for ^64^Cu from endogenous proteins that are able to strongly chelate copper.^60^, ^62^ NOTA chelators have been found to largely circumvent this issue, forming much more stable copper complexes within shorter labelling times, displaying decreased nonspecific uptake in the liver and spleen.^63^, ^64^ For these reasons there has been a shift toward using NOTA as the chelator of choice for ^64^Cu‐labelling of fragments. Cai and co‐workers^65^ recently reported the ^64^Cu‐labelling of a bispecific fragment using a NODA chelator and used it for imaging CD105 and tissue factor (TF) in mice bearing pancreatic cancer xenographs. The same fragment was also labelled with a fluorescent tag, to generate a dual labelled probe, that also enabled near‐infrared fluorescence (NIRF) imaging. The labelled heterodimer probe showed increased tumour uptake relative to the homodimer and the fluorescence imaging was used to validated the PET imaging and to show delineation of the tumours. Such dual‐modality optical‐PET imaging is advantageous over single modality imaging, as the much higher spatial resolution of optical imaging methods relative to PET can aid in more accurately localising the probe. Dual optical‐PET and optical fragment‐based probes may further help in the diagnosis and staging of cancer, as well as in providing greater delineation of healthy and cancerous tissue during surgery.^66^, ^67^, ^68^

**Figure 5:**
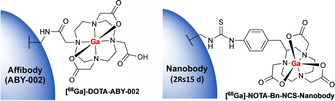
Cyclic chelators DOTA^14^, ^72^, ^73^, ^74^ and NOTA^75^, ^76^, ^77^ that have been conjugated to antibody fragments or affibodies and radiolabelled with gallium‐68.

Recently, shorter‐lived radiometals such as ^68^Ga (*t*_1/2_=67.7 min) and ^44^Sc (*t*_1/2_=3.9 h), with half‐lives more closely matching the pharmacokinetics of antibody fragments, have garnered closer attention for fragment labelling. ^68^Ga is particularly convenient for radiolabelling because it is produced via a ^68^Ge/^68^Ga generator and thus circumvents the need (and associated costs) of a cyclotron facility. A common approach for ^68^Ga‐labelled is reaction with the widely used DOTA and NOTA,^69^ however, a range of other bifunctional chelators have been investigated.^70^ The FDA approved ^68^GaDOTA‐TATE tracer for somatostatin receptor positive neuroendocrine tumours is an example of its growing importance for clinical use.^71^ ^68^Ga‐DOTA labelling, however, typically requires elevated temperatures to ensure high yielding and rapid incorporation; therefore direct labelling of heat sensitive proteins with ^68^Ga is not typically achieved with DOTA chelators. NOTA chelators are more suitable for direct ^68^Ga‐labelling of antibody fragments. Both the thermodynamic stability Ga^III^‐NOTA complexes (log*K*=31.0) and the kinetics of chelation are superior to that of Ga^III^‐DOTA (log*K*=21.3).^70^ ^68^Ga‐NOTA labelling can therefore be achieved at room temperature within much shorter reaction times to generate complexes that are typically much more stable in vivo. A range of Fabs, nanobodies and affibodies have been successfully labelled with ^68^Ga using the cyclic DOTA^14^, ^72^, ^73^, ^74^ and NOTA^75^, ^76^, ^77^ chelators (Figure 5). Recently, a HER2 specific ^68^Ga‐NOTA‐Bn‐SCN‐Nanobody underwent phase I clinical trials and displayed high uptake in metastases.^76^

**Figure 6:**
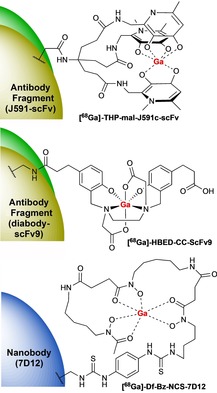
The acyclic chelators THP, HBED and DFO that have been conjugated to antibody fragments and radiolabelled with gallium‐68.^79^, ^80^, ^81^, ^82^

Desferrioxamine B (DFO), an acyclic chelator and naturally occurring siderophore, has been widely used to radiolabelled antibodies. The acyclic and multidentate nature of this ligand enables the rapid and stable formation of Ga‐chelates under mild reaction conditions, typically within 5 min at room temperature. Several ^68^Ga‐DFO nanobody and scFv fragments have been reported for imaging EGFR and HER2 (Figure 6).^78^, ^79^ There are, however, some concerns over the stability of ^68^Ga‐DFO complexes and in their labelling efficiencies relative to ^68^Ga‐NOTA complexes.^79^ *N*,*N*′‐Bis(2‐hydroxybenzyl)ethylenediamine‐*N*,*N*′‐diacetic acid (HBED) is another acyclic chelator that enables rapid ^68^Ga‐labelling at room temperature. The HBED‐CC derivative has been used for rapid and room temperature labelling of recombinant scFvs and diabodies (Figure 6).^80^, ^81^ A tris(hydroxypyridinone) (THP) bifunctional chelator has recently been used to label scFv of J591, (Figure 6) for prostate‐specific membrane antigen (PSMA) imaging.^82^ The THP derivative (THP‐mal) was coupled the C‐terminus of a cysteine residue of the scFv via a maleimide linker and labelled ^68^Ga at room temperature and neutral pH achieving RCY >95 % without further purification. The THP ligand system is proving to be a highly effective chelator for ^68^Ga that shows excellent stability.^83^

Zirconium‐89 (*t*_1/2_≈78 h) is a longer lived PET radionuclide that has found applications for the labelling of intact antibodies and for larger antibody fragments such as minibodies and diabodies. The DFO ligand is commonly used to chelate ^89^Zr^IV^, however, studies have demonstrated ^89^Zr‐DFO complexes to be unstable in vivo, resulting in ^89^Zr release and accumulation of ^89^Zr in bone.^23^ Despite this, DFO has been mostly used for fragment labelling due to its high chelation yields, mild reaction conditions and reasonable stabilities. Wu and Lewis recently reported the ^89^Zr labelling of PMSA selective minibody and diabody fragments.^84^ Labelling was achieved in both cases with good RCY (>70 %) via chelation with DFO modified fragments. In comparison with the intact antibody, also labelled with ^89^Zr‐DFO, both fragments displayed faster tumour delineation and background clearance in tumour‐bearing mice, thus demonstrating their potential as probes for the detection and staging of PSMA‐positive tumours. A [^89^Zr]ZrDFO‐Cys‐diabody has recently been developed for tracking endogenous CD8^+^ T‐cells which could be used to evaluate the tumour immune response of novel immunotherapies.^85^ The Cys‐diabody was conjugated to a maleimide‐DFO via the engineered C‐terminal cysteine of the fragment and labelled with ^89^Zr in high RCY. The labelled diabody showed specific targeting to CD8 in tumour‐bearing mice. An interesting nanobody construct, composed of three individual nanobodies, that is capable of binding albumin and two different epitopes of HER3 has also been labelled via ^89^Zr via DFO chelation.^86^ It has been previously found that two anti‐HER3 antibodies inhibited tumour cell growth better than each antibody independently. The labelled construct showed an uptake correlation with HER3 expression in tumour‐bearing mice, and enabled tumour visualisation for up 96 h post injection. A ^89^Zr‐DFO labelled minibody fragment has recently undergone a phase I clinical trial for imaging metastatic prostate cancer.^87^ The labelled minibody proved to be safe and showed favourable biodistribution for imaging metastatic prostate cancer. The limitations of ^89^Zr‐labelling, include the low positron abundance (23 %) and long half‐life which mean that patients are potentially exposed higher doses of radioactivity. Recent studies have demonstrated that ^89^Zr‐DOTA derivatives show greater stability, however, chelation requires heating at >90 °C which could not be applied to the direct labelling with fragments.^88^

**Scheme 9:**
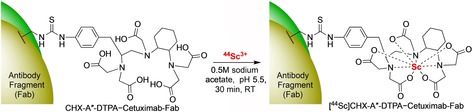
^44^Sc‐labelling of a Fab fragment using a thiourea‐conjugated DTPA chelator.^94^

Scandium‐44 is receiving current interest owing to its intermediate 3.97 h half‐life, high positron yield, route of production and coordination chemistry.^89^ It is typically produced from ^44^Ca in a cyclotron,^90^ but has the potential to be more conveniently produced via a ^44^Ti/^44^Sc generator system.^91^ ^44^Sc, which is considered to be a potential alternative to ^68^Ga, displays similar coordination chemistry to ^68^Ga and could be used to extend the PET scanning window which may result in better images for certain probes.^92^ The longer 3.97 h half‐life is particularly well matched for antibody fragment imaging studies. The vast majority of ^44^Sc labelling has been focused on DOTA functionalised peptides, however, chelation of Sc^III^ to DOTA requires high temperature due to its slow reaction kinetics which is unfavourable for protein labelling. An affibody molecule, Z_HER2:2891_, has been recently labelled with ^44^Sc using an N‐terminal conjugated DOTA chelator.^93^ High RCY were achieved via reaction with ^44^ScCl_3_ at 95 °C for 30 min. The [^44^Sc]Sc‐DOTA‐Z_HER2:2891_ conjugate is promising and displays specific binding to HER2‐expressing cells, and high‐contrast for the imaging of tumour‐bearing mice. Cai and co‐workers^94^ reported ^44^Sc labelling of a cetuximab Fab fragment modified with a diamine‐pentaacetic acid (DTPA) chelator ([^44^Sc]CHX‐A′′‐DTPA‐cetuximab‐Fab, Scheme 9) which enabled radiolabelling within 30 min, under optimised conditions. The labelled fragment displayed good stability in mouse serum, specific uptake in tumour‐bearing mice and rapid renal clearance.

## 4 Summary and Outlook

A range of PET radionuclides have been used to generate an array of labelled fragments and affibodies for imaging studies; key labelling methods, targets and imaging applications are summarised in Tables 2 and 3. The short‐to‐medium lived radionuclides ^18^F, ^68^Ga, ^124^I and ^64^Cu are currently the most widely used for fragment labelling studies owing to their availability and half‐lives that match the biological clearance rates of fragments. Labelling methods have evolved considerably over the past ten years. There is now a diverse range of prosthetic groups and conjugation methods that can be used to reliably label fragments, although there is room for improvement in terms of labelling timeframes, radiochemical yields and simplification of labelling procedures. Key considerations for fragment labelling include matching the radionuclide to the pharmacokinetics of the fragment, labelling rapidly under mild conditions to ensure fragment integrity and labelling at specific sites within the fragment to ensure that the immunoreactivity of the fragment with its target is not compromised. Lysine and cysteine bioconjugation methods are now well‐established for fragment and affibody labelling. Newer bioconjugation strategies, such as the IEDDA reaction (using Tz/TCO), offer improved rates of reaction under milder conditions but require more complex synthetic steps to prepare appropriate fragment conjugates and labelling partners. Improved chelator chemistry has also evolved for rapid and mild sequestering of radiometals. THP and NOTA derivatives are two key examples of chelators that show the greatest kinetic and thermodynamic stabilities, in addition to mild and rapid chelation reaction conditions. The establishment of novel labelling routes such as the [^18^F]AlF labelling, greater access to existing PET radionuclides (e.g., generator produced ^68^Ga) and access to new radionuclides with favourable physical characteristics for labelling (e.g., ^44^Sc) have also enabled the development of fragment‐based imaging agents. As new fragments are developed for both therapeutic and imaging, new mild, rapid, site‐specific and robust labelling methods will aid in the understanding of their biological behaviour and facilitate clinical translation. Inevitably, research in fundamental chemistry and its translation can profoundly impact imaging chemistry. The development of new bioconjugation and chelation methods are likely to be key to improving fragment labelling protocols, and ultimately impact clinical use.

**Table 2:** Summary of targets and imaging applications of PET radiolabelled antibody fragments and affibodies.

Target	Imaging applications
human epidermal growth factor receptor 1 (EGFR or HER1)	glioblastoma multiforme (GBM); head and neck squamous cell carcinoma (HNSCC); breast cancer; non‐small‐cell lung cancer (NSCLC)
human epidermal growth factor receptor 2 (HER2)	breast cancer; ovarian cancer; lung cancer; gastric cancer
human epidermal growth factor receptor 3 (HER3)	resistance to EGFR and HER2 therapies; HNSCC; NSCLC
epidermal growth factor receptor variant III (EGFRvIII)	glioblastoma multiforme (GBM)
αvβ6 integrin	oral squamous cell cancer (SCC); pancreatic cancer; ovarian cancer; cervical cancer
endoglin (CD105)	angiogenesis; pancreatic cancer
tissue factor (TF)	angiogenesis; pancreatic cancer; triple‐negative breast cancer (TNBC); abdominal aortic aneurysm (AAA)
epithelial membrane protein‐2 (EMP2)	endometrial cancer; ovarian cancer
C‐kit	gastrointestinal stromal tumours (GIST); small‐cell lung cancer (SCLC)
glycoprotein 72 (TAG‐72)	breast cancer; ovarian cancer; lung cancer; colon cancer; pancreatic cancer
ERC/mesothelin	mesothelioma; ovarian cancer
CD8+ T‐cells	immune response; cancer immunotherapy
CD4+ T‐cells	immune response; inflammatory bowel disease
B‐cell differentiation antigen (CD20)	non‐Hodgkin lymphoma (NHL)
prostate‐specific membrane antigen (PSMA)	prostate cancer; metastatic prostate cancer
CA125	epithelial ovarian cancer (EOC); breast cancer; mesothelioma; leiomyoma
major histocompatibility complex (MHC) class II	pancreatic cancer
urokinase plasminogen activator receptor (uPAR)	non‐small‐cell lung cancer (NSCLC); colon cancer (CRC)
epithelial cell adhesion molecule (EpCAM)	solid tumours
vascular cell adhesion molecule (VCAM‐1)	atherosclerosis

**Table 3:** Summary of selected ^18^F, ^124^I, ^11^C, ^64^Cu, ^68^Ga, ^89^Zr and ^44^Sc‐radiolabelled antibody fragments and affibodies, and their targets.

PET nuc.	Prosthetic group/reagent/chelator‐linker	Antibody fragment or affibody	Target	References
^18^F	[^18^F]BMEM	Z_HER2:342_ affibody	HER2	35
^18^F	[^18^F]SFB	Cys‐diabody	HER2	9
^18^F	[^18^F]SiFA, [^18^F]AlF‐NOTA‐MPAEM, and [^18^F]FBA	Z_HER2:2891_	HER2	36, 37
^18^F	[^18^F]AlF(RESCA)	PEP04314 affibody	HER2	44
^18^F	[^18^F]AlF‐NODA‐Tz	Z_HER3:8698_ affibody	HER3	43
^18^F	[^18^F]AlF‐NODA‐MPAEM	hMN‐14‐(scFv)2 (diabody); hMN‐14 Fab‐AD2	CEA	42
^18^F	[^18^F]SFB	scFv‐B43.13	CA125	97
^18^F	[^18^F]FDG‐Tz	V_H_H7 nanobody	MHC class II	40
^18^F	[^18^F]FA (enzymatic)	2G10‐Fab	uPAR	45
^18^F	[^18^F]SFB	cAbVCAM‐1‐5 nanobody	VCAM‐1	98
^124^I	[^124^I]NaI, Iodogen	rituximab minibody (scFv‐CH3 dimer); scFv‐Fc	CD20	48
^124^I	[^124^I]NaI, Iodogen	rituximab Cys‐diabody	CD20	49
^124^I	[^124^I]NaI, Iodogen; SHPP method	C6.5 diabody	HER2	50
^124^I	[^124^I]NaI, chloramine‐T, SPIB method	Z_HER2:342_ affibody	HER2	52
^124^I	[^124^I]NaI, Iodogen	T84.66 single‐chain Fv‐Fc	CEA	99
^124^I	[^124^I]NaI, Iodogen	T84.66 minibody; T84.66 diabody	CEA	100
^124^I	[^124^I]NaI, Iodogen	A11 anti‐PSCA minibody	PSCA	101
^124^I	[^124^I]NaI, Iodogen	2B3 minibody	PSCA	102
^11^C	[^11^C]methyl iodide; Sel‐Tag	Z_HER2:342_ affibody	HER2	53
^11^C	l‐[^11^C]methionine	scFv (MR1‐1)	EGFRvIII	54
^64^Cu	DOTA‐GLGK hexavinylsulfone	anti‐CEA‐diabody	CEA	29
^64^Cu	DOTA‐NHS‐ester	rituximab minibody (scFv‐CH3 dimer); scFv‐Fc	CD20	48
^64^Cu	DOTA‐NHS‐ester	T84.66 minibody	CEA	56
^64^Cu	DOTA‐NHS‐ester	AVP04‐07 diabody	TAG‐72	57
^64^Cu	DOTA‐Bn‐NCS	12A8 Fab	C‐kit	58
^64^Cu	DOTA‐Bn‐NCS	anti‐C‐ERC Fab	ERC/mesothelin	59
^64^Cu	DOTA‐NHS‐ester	anti‐EMP2 minibody	EMP2	60
^64^Cu	NOTA‐Bn‐NCS	Fab′′ heterodimer (CD105/TF); ALT‐836‐Fab	TF	65, 96
^64^Cu	NOTA‐Bn‐NCS	bispecific‐F(ab)_2_	EGFR, HER1	103
^64^Cu	NOTA‐maleimide	αvβ_6_ Cys‐diabody	αvβ6 integrin	104, 105
^64^Cu	NOTA‐Bn‐NCS	Fab	CD105	106
^68^Ga	DOTA‐N‐terminus	ABY‐025 affibody; F(ab′)_2_‐trastuzumab; Z_HER2:342_ affibody (ABY‐002)	HER2	14, 72, 73, 74
^68^Ga	NOTA‐Bn‐NCS	anti‐HER2‐nanobody (2Rs15 d); panitumumab F(ab′)_2_	HER2	75, 76, 77
^68^Ga	DFO‐Bn‐NCS	nanobody 7D12	EGFR/HER1	79
^68^Ga	HBED‐CC	recombinant antibody fragments scFv	EpCAM	80
^68^Ga	HBED‐CC	diabody scFv9 anti Ep‐CAM	EGFR/HER1	81
^68^Ga	THP‐mal	J591 scFv	PSMA	82
^89^Zr	DFO‐*N*‐succinyl	MSB0010853 (nanobody)	HER3	86
^89^Zr	DFO‐maleimide	anti‐CD8 Cys‐diabody	CD8+ T‐cells	85
^89^Zr	DFO‐maleimide	anti‐CD4 Cys‐diabody	CD4+ T‐cells	5, 107
^89^Zr	DFO‐Bn‐NCS	huJ591‐minibody; huJ591‐Cys‐diabody	PSMA	84
^89^Zr	DFO‐*N*‐succinyl	IAB2M minibody	PSMA	87
^89^Zr	DFO‐maleimide	Z_EGFR:2377_ (affibody)	EGFR/HER1	108
^89^Zr	DFO‐*N*‐succinyl	A11 anti‐PSCA minibody	PSCA	101
^44^Sc	DOTA‐Bn‐NCS	Z_HER2:2891_ (affibody)	HER2	93
^44^Sc	DTPA‐CHX‐A′′‐	cetuximab‐Fab	EGFR/HER1	94

The development of labelled fragments and affibodies is without doubt a long, challenging and expensive task that requires iterative stages of protein engineering, selection of a fragment/affibody, appropriate radiolabelling and preclinical testing. For clinical translation there are regulatory hurdles and challenges in scaling‐up of the fragment/affibody production process. The timeframes and cost of developing a novel antibody fragment/affibody imaging agent may, however, be reduced compared with full antibody agents, due to their more rapid production, selection and characterisation methods. There is enormous potential and many opportunities for imaging with antibody fragments and affibodies based on their high affinities, specific targeting and fast clearance rates. The non‐invasive imaging of cell‐surface antigens for cancer detection has been the main focus to date and there has been a justified emphasis on targeting EGFRs (HER1‐HER3) owing to their over‐expression in a wide range of cancers. Future challenges in this area involve improving the specificity of fragment‐based probes to the HER family in order to better select patients for receptor‐targeted therapy and monitoring of therapeutic response.^95^

Encouragingly, several labelled fragments are now undergoing clinical trials as cancer imaging agents.^76^, ^87^ A number of other targets have been investigated (Tables 2 and 3), and there is much current interest in imaging inflammatory responses via the direct targeting of T‐cells. It is also expected that labelled fragments will be used to specifically target antigens of pathogenic bacteria and viruses, and thus enable the development of new pathogen‐specific tracers to discriminate between infectious and sterile sites of inflammation. Future developments in this area will depend on the identification of new fragments and affibodies that have appropriate characteristics for imaging (high target affinity, selectivity, stability, rapid clearance, ease of labelling etc.). The adoption of newer and more site‐specific conjugation chemistries is currently underway, as is the development of theranostic fragments that will make use of complementary radionuclide pairs for both imaging and therapy (e.g., ^44^Sc/^47^Sc, ^124^I,^131^I). It is also anticipated that PET imaging will play a role in the assessment of the burgeoning field of fragment‐based antibody drug conjugates (ADCs).

## Conflict of interest

*The authors declare no conflict of interest*.
